# Quantitative proteomics screen identifies a substrate repertoire of rhomboid protease RHBDL2 in human cells and implicates it in epithelial homeostasis

**DOI:** 10.1038/s41598-017-07556-3

**Published:** 2017-08-04

**Authors:** Nicholas Johnson, Jana Březinová, Elaine Stephens, Emma Burbridge, Matthew Freeman, Colin Adrain, Kvido Strisovsky

**Affiliations:** 1 Institute of Organic Chemistry and Biochemistry, Czech Academy of Science, Flemingovo n. 2, Prague, 166 10 Czech Republic; 2 Department of Biochemistry, Faculty of Science, Charles University, Prague, Czech Republic; 3 MRC Laboratory of Molecular Biology, Cambridge, CB2 2QH United Kingdom; 4 Sir William Dunn School of Pathology, Oxford, OX1 3RE United Kingdom; 5 Instituto Gulbenkian de Ciência, Lisbon, Portugal

## Abstract

Rhomboids are intramembrane serine proteases conserved in all kingdoms of life. They regulate epidermal growth factor receptor signalling in *Drosophila* by releasing signalling ligands from their transmembrane tethers. Their functions in mammals are poorly understood, in part because of the lack of endogenous substrates identified thus far. We used a quantitative proteomics approach to investigate the substrate repertoire of rhomboid protease RHBDL2 in human cells. We reveal a range of novel substrates that are specifically cleaved by RHBDL2, including the interleukin-6 receptor (IL6R), cell surface protease inhibitor Spint-1, the collagen receptor tyrosine kinase DDR1, N-Cadherin, CLCP1/DCBLD2, KIRREL, BCAM and others. We further demonstrate that these substrates can be shed by endogenously expressed RHBDL2 and that a subset of them is resistant to shedding by cell surface metalloproteases. The expression profiles and identity of the substrates implicate RHBDL2 in physiological or pathological processes affecting epithelial homeostasis.

## Introduction

Proteins of the rhomboid family are the most widely occurring intramembrane proteases and are spread throughout the tree of life. Rhomboid proteases are cardinal regulators of EGF receptor signalling in *Drosophila* ^1^ but their functions in mammals are poorly understood; no mouse knockout experiments have been published for the human non-mitochondrial rhomboids and their substrate repertoires are largely unknown. In mammals, four rhomboid proteases (RHBDL 1–4) localize to the secretory pathway^2, 3^. The endoplasmic reticulum (ER) localized RHBDL4 (also known as RHBDD1) may be involved in ER associated degradation^3^, oncogenic signalling^4^ and regulation of ER export of transmembrane cargoes^5^, but the major substrate(s) involved are unknown or disputed^2, 3^. No substrates have yet been identified for RHBDL1 and 3; hence although they exhibit all the sequence hallmarks of active proteases^6^, their proteolytic activity has not yet been formally demonstrated. As understanding a protease’s substrate repertoire is the key to unveiling its functions, a significant bottleneck in the field is the lack of effective strategies for rhomboid protease substrate identification.

The best understood mammalian ‘secretase’ rhomboid thus far is RHBDL2, which localizes to the plasma membrane^2^ where it can catalyse proteolysis of cell surface transmembrane proteins. Current knowledge of rhomboid protease substrates is largely based upon candidate screening approaches and experimentation with heterologous substrates. Candidate screens have identified several substrates of RHBDL2, such as B-type ephrins^7^, which act as ligands for Eph tyrosine kinase receptors, thrombomodulin^2^, a cell-surface membrane protein involved in the regulation of blood coagulation, epidermal growth factor (EGF)^8^, and C-type lectin CLEC14A^9^. However, at least thrombomodulin^10^, B-type ephrins^11^ and EGF^12^ can also be shed by ADAM metalloproteases, the major ectodomain-shedding enzymes on the surface of mammalian cells, suggesting redundancy between rhomboids and ADAMs. Overall, the lack of systematic and objective approaches to identify rhomboid substrates severely limits our ability to understand the physiological roles of rhomboid proteases.

To address these shortcomings, in this study we use quantitative proteomics to objectively identify the substrate repertoire of RHBDL2. We find that a range of type I membrane proteins are specifically cleaved and shed into the media upon co-expression with RHBDL2. These include the cell surface protease inhibitor Spint-1 (also known as HAI-1); the receptor tyrosine kinase DDR1; the receptor for interleukin-6 (IL6R), and the cell surface proteins CLCP1 (also known as DCBLD2 or ESDN) and KIRREL (also known as Neph1). Notably, the shedding of these novel substrates is specific: they are not cleaved by other human RHBDLs, and the substrates are largely resistant to shedding by the major ADAM metalloproteases, ADAMs −10 and −17. We demonstrate endogenous activity of RHBDL2 in cell lines, reinforcing the physiological relevance of these observations. Based on the identified substrate repertoire and their expression patterns, we propose that RHBDL2 functions in epithelial homeostasis, potentially in the skin, airways or digestive system. Our data indicate that mammalian rhomboids have a specific substrate repertoire and can function autonomously from metalloproteases, in distinct signalling pathways.

## Results

### Quantitative proteomics identifies substrate repertoire of RHBDL2 in human cells

To identify endogenous substrates of RHBDL2, we carried out an overexpression screen in HEK293ET cells, which do not express endogenous RHBDL2^8^, thus constituting a sensitive environment for the readout of RHBDL2 activity. We chose to use stable isotope labelling in cell culture (SILAC)^13^, an approach that involves growing two (or more) experimental cellular populations in medium containing heavy versus light isotopes of essential amino acids (typically Arg and Lys), which results in uniform isotopic labelling of whole proteomes. The advantage of this technique is that different experimental samples can be pooled early in analysis, hence, any fractionation and enrichment procedures affect all conditions equally, minimising variation. This was important because we envisaged using enrichment of the secretome, and subcellular membrane fractionation, which can introduce significant bias between samples. The isolated protein mixture can then be interrogated by mass spectrometry, allowing quantitative comparison of the heavy or light isotopic versions of the otherwise identical peptides, eventually allowing comparison of the relative abundance of individual proteins between different experimental conditions.

**Figure 1:**
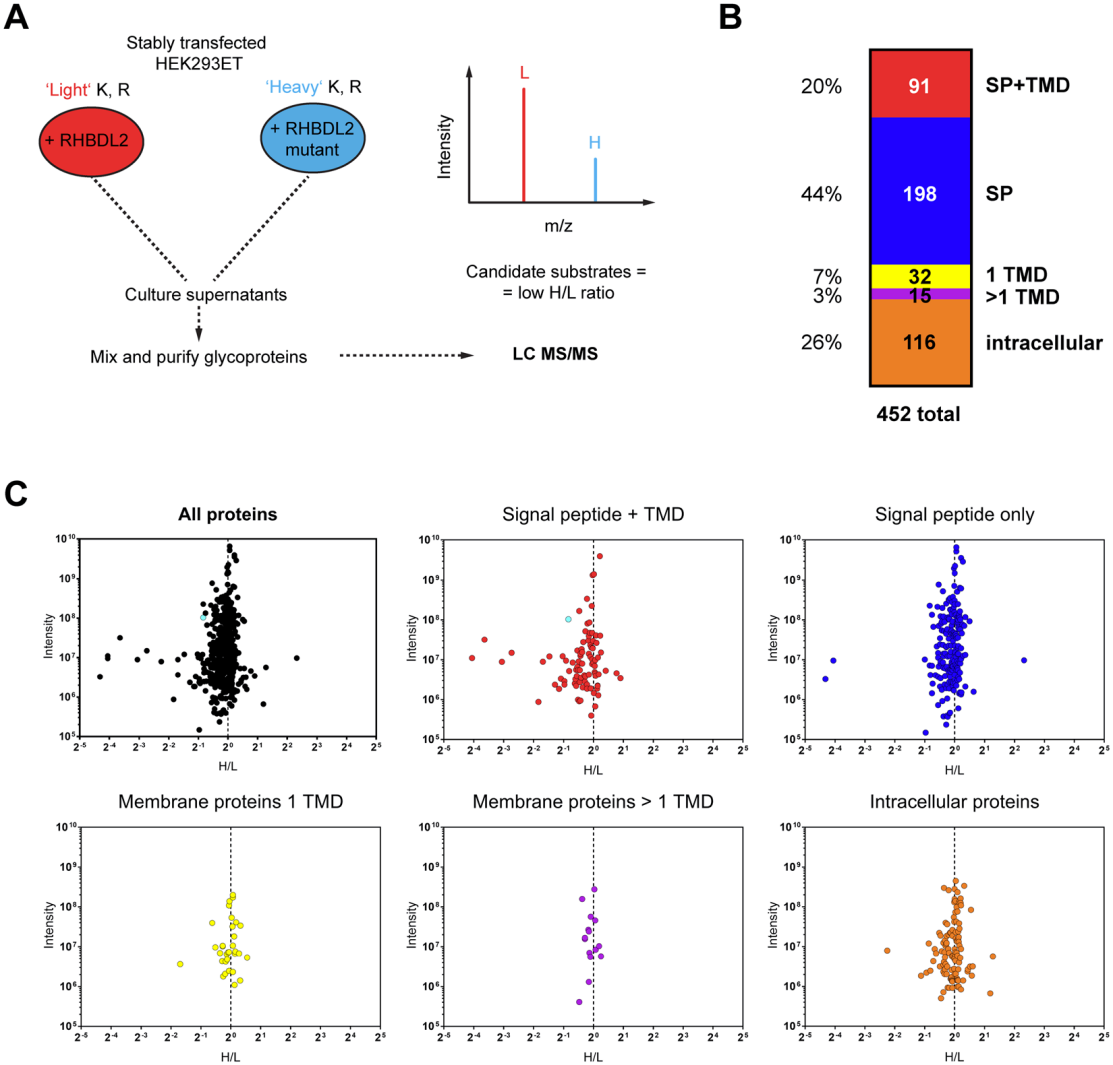
Quantitative proteomics screen to identify substrate repertoire of RHBDL2. (**A**) HEK cells stably expressing either active RHBDL2 or an inactive mutant were cultured with unmodified (‘light’) arginine and lysine or those bearing ‘heavy’ isotope labels (see Materials and methods for details), respectively. The conditioned media from both cultures were pooled and glycoproteins enriched by lectin affinity chromatography. Following enzymatic digestion and fractionation, peptides were analysed by mass spectrometry. Proteins in which the unlabelled (light) form predominates over the isotopically labelled (heavy) form can be considered candidate substrates of RHBDL2. (**B**) Proteins identified in the secretome were grouped by their predicted localization and topology. (**C**) Analysis of the secretome reveals a significant enrichment in proteins of heavy to light (H/L) ratios significantly lower than 1 manifesting as an asymmetric plot of total ion intensity per protein over its H/L ratio (black data points). A breakdown into the different topological groups as classified and colour coded in panel B reveals that most of the asymmetry is due to type I membrane proteins. The cyan dot is the positive control, the validated substrate mouse EGF^8^.

To create cell lines suitable for the SILAC analysis, HEK293ET cells were first stably transduced with lentiviruses expressing EGF, a known RHBDL2 substrate, hence providing an internal positive control RHBDL2 substrate. These EGF-expressing cells were subsequently transduced with lentiviruses expressing either RHBDL2 or its catalytically dead serine to alanine catalytic site mutant, to achieve stable constitutive expression. To compare the secretomes of these two cell populations and thus identify the substrate repertoire of RHBDL2, both cell lines were labelled by SILAC and their extracellular proteomes were compared quantitatively (Fig. 1A). To suppress the signal of the residual serum proteins and of intracellular proteins resulting from occasional cell lysis, the secreted and cell surface proteins were enriched by lectin affinity chromatography.

Analysis of the purified secretomes by mass spectrometry identified 452 quantifiable proteins in total (excluding contaminants). Of these, 44% were secreted proteins, 20% were type I membrane proteins (i.e. proteins that originate with a signal peptide and transmembrane domain^14^), 10% comprised other membrane proteins and the remaining 26% were intracellular proteins (Fig. 1B). We next scrutinized the dataset for candidate RHBDL2 substrates. As we grew the cells expressing WT RHBDL2 in SILAC medium containing a light SILAC isotope, whereas the cells expressing the catalytically dead mutant were grown in heavy isotopic medium, candidate RHBDL2 substrates, secreted into the culture medium should exhibit a heavy-to-light (H/L) ratio of less than 1.0. The list of all identified proteins, ranked by their H/L ratio, is shown in Table S1.

As anticipated, plotting the total ion intensity against relative abundance for all identified and quantified proteins reveals a significant asymmetry towards the low H/L ratios (Fig. 1C, top left panel) indicative of proteins that were potential RHBDL2 substrates. When all identified proteins were grouped according to their membrane topology, predicted by the Phobius algorithm^15^, it became clear that most of the asymmetry in the intensity to abundance plot was due to type I membrane proteins (Fig. 1C). Interestingly, most known rhomboid protease substrates fall into this topological category, suggesting that these proteins could be true RHBDL2 substrates. Indeed, the cohort of proteins with an H/L less than 1 included our positive control substrate, EGF (marked in cyan). EGF had an H/L ratio of 0.56, that is, approximately twice the amount of cleaved EGF could be detected in the secretome from cells expressing wild type RHBDL2 than its catalytic mutant. Interestingly, 12 other type I membrane proteins displayed H/L ratios lower than that of EGF, indicating that these proteins might have been released into the medium by the activity of RHBDL2. To validate the results of this proteomic analysis we selected the 12 type I membrane proteins with H/L ratio lower than that of EGF, and 9 more type I membrane proteins spanning a range of H/L ratios up to around 1 for further validation and analysis (Table 1).

**Table 1:** Type I membrane proteins identified by SILAC screen for substrates of RHBDL2 that were selected for validation.

H/L	Uniprot accession	Protein names	Common names	Length	Phobius SP	Phobius TMD	Uniprot TMD	Topology prediction	Fragment tested
0.06	P50895	Basal cell adhesion molecule	BCAM	628	Y	1	1	n16-26c31/32o549-571i	aa32-628
0.08	P19022	Cadherin-2, Neural cadherin	Cdh2 (Ncadh)	906	Y	1	1	n10-20c25/26o724-746i	aa26-906
0.12	O43278	Kunitz-type protease inhibitor 1	Spint1	529	Y	1		n19-30c35/36o466-488i	aa36-529
0.15	^$^AAH47021.1	Cell adhesion molecule 1	Cadm1	387	Y	1	1	n4-14c19/20o347-372i	aa20-387
*0.*18*	*Q08345-1*	*Epithelial discoidin domain-containing receptor 1*	*DDR1*	*913*	*Y*	*1*	*1*	*n5-15c20/21o417-439i*	*aa21-913*
0.28	P35613	Basigin	BSG	385	Y	1	1	n3-14c21/22o324-345i	aa22-385
0.31	Q96PD2-2	Discoidin, CUB and LCCL domain-containing protein 2	DCBLD2/CLCP1/ESDN	789	Y	3	1	^#^n20-30c66/67o529-549i	aa67-789
0.36	P52799	Ephrin-B2	EFNB2	333	Y	1	1	n12-20c25/26o225-250i	aa28-333
0.41	Q96J84	Kin of IRRE-like protein 1	KIRREL	757	Y	1	1	n2-11c16/17o495-521i	aa17-757
0.51	P98172	Ephrin-B1	EFNB1	346	Y	1	1	n13-24c29/30o238-263i	aa30-346
0.51	Q86XX4-2	Extracellular matrix protein FRAS1	FRAS1	4012	Y	1	1	n6-14c26/27o3906-3926i	aa3775-4012
0.53	Q9UK23	N-acetylglucosamine-1-phosphodiester alpha-N-acetylglucosaminidase	UCE	515	Y	1	1	n8-19c24/25o449-473i	aa25-515
**0.56**	**P01132**	**Pro-epidermal growth factor**	**EGF**	**1217**	**Y**	**1**	**1**	**n8-19c28/29o1039-1063i**	**aa28-1217**
0.57	P01130	Low-density lipoprotein receptor	LDLR	860	Y	1	1	n11-19c24/25o789-810i	aa25-860
0.68	Q13444	Disintegrin and metalloproteinase domain-containing protein 15	ADAM15	863	Y	1	1	n3-14c22/23o694-717i	aa18-863
0.69	O75976	Carboxypeptidase D	CPD	1380	Y	1	1	n16-26c31/32o1300-1323i	aa217-1380
0.72	P08887	Interleukin-6 receptor subunit alpha	IL6R	468	Y	1	1	n3-14c19/20o367-388i	aa20-468
0.90	Q14703	Membrane-bound transcription factor site-1 protease	S1P	1052	Y	1	1	n6-17c22/23o1002-1022i	aa18-1052
0.97	P12821	Angiotensin-converting enzyme	ACE	1306	Y	1	1	n11-22c29/30o1260-1281i	aa30-1306
0.98	Q92896	Golgi apparatus protein 1	GLG1	1179	Y	1	1	n13-23c27/28o1146-1169i	aa44-1179
0.99	O15031	Plexin-B2	PLXNB2	1838	Y	1	1	n3-14c18/19o1200-1221i	aa800-1838
1.03	O95185	Netrin receptor UNC5C	UNC5C	931	Y	1	1	n12-31c39/40o377-402i	aa40-931

**Figure 2:**
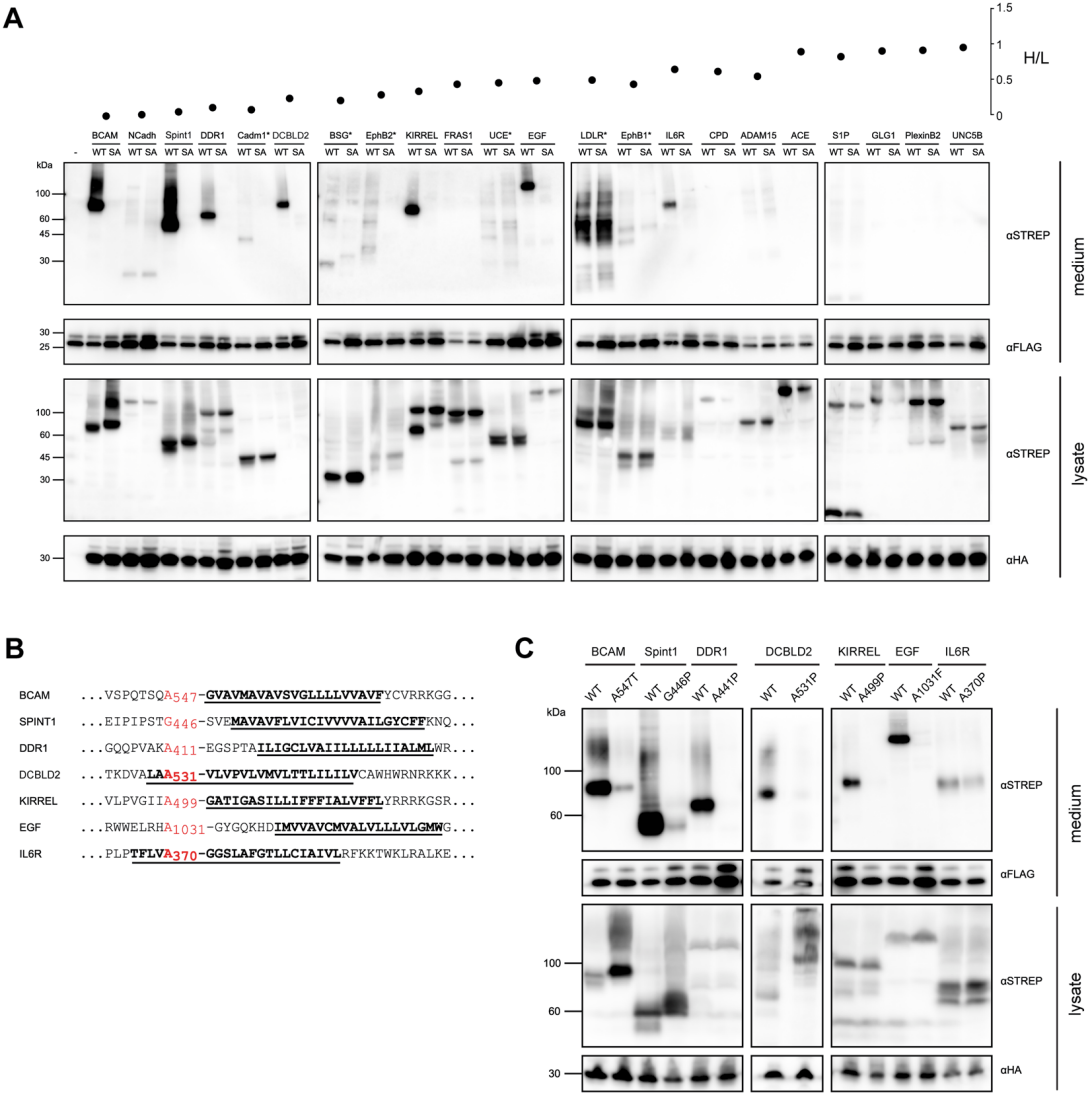
Validation of candidate substrates of RHBDL2 identified by SILAC proteomics screen (**A**). A selection of Strep tagged proteins identified by the SILAC screen, of increasing heavy to light ratio, were co-expressed with HA-tagged forms (lower lysate panel, αHA) of either wild-type mouse RHBDL2 (WT) or an inactive mutant in which the catalytic serine residue was mutated to an alanine (SA). Twenty four hours after transfection the media were replaced by serum free media containing a broad spectrum matrix metalloprotease inhibitor (BB94) to exclude shedding by these extracellular proteases. After 24 hours the media and cell lysates were harvested and analysed by immunoblotting for the tagged proteins using anti-Strep. Cells were co-transfected with FLAG tagged prolactin to act as a secretion control and to confirm equal recovery of TCA precipitated proteins from the media (lower media panel, αFLAG). (**B**) Summary of cleavage sites of RHBDL2 substrates identified by mass spectrometry of purified ectodomain fragments. The P1 residue is highlighted in red and the TMD is underlined and highlighted in bold. (**C**) To confirm the cleavage site, the P1 residue was mutated to a residue typically not accepted in the P1 position of rhomboid substrates, such as proline, threonine or phenylalanine^8, 17, 18^. The wild-type and mutant forms of each substrate were subsequently co-expressed with active RHBDL2 in a cellular cleavage assay as described in Fig. 1. Conditioned media (upper panels) were immunoblotted for Strep tagged substrates and FLAG tagged prolactin and cell lysates (lower panels) immunoblotted for Strep tagged substrates and HA tagged RHBDL2 respectively.

The cleavage of proteins displayed in Fig. 2 was further validated. The topology prediction is notified as used by the Phobius predictor^15^, that is ‘n’ for the N-terminus, ‘/’ for signal peptide cleavage site, ‘o’ for outer and ‘i’ for inner localisation with respect to the plasma membrane; hyphenated number ranges denote positions of transmembrane helices or signal peptides. ^$^This is NCBI protein record AAH47021.1, which corresponds to the cDNA IMAGE clone number 5248100 sequence used in this work (which is identical to the NCBI nucleotide record BC047021.1). ^#^For DCBLD2/CLCP1, Phobius predicts 3 TMHs, but the protein is known to have only 1 TMH, and its real topology derived from Uniprot entry Q08345 is displayed. *DDR1 (in italics) was identified only in the initial database searches and not using the current Uniprot/Swissprot human database containing protein isoforms (downloaded 17/03/03), but since it was meanwhile confirmed as a substrate, it has been included also in this final data table. The fragment of each individual protein cloned into N-terminally Strep-His tagged constructs for validation of the SILAC results are denoted by amino acid (aa) ranges.

This subset of 22 proteins (including EGF) with H/L ratios ranging from 0.06 to 1.03 were tested for cleavage by RHBDL2 in co-expression experiments in mammalian cells. The tested proteins were tagged at the N-terminus with tandem One-Strep^16^ and His tags, and co-expressed with RHBDL2 or its catalytic serine to alanine mutant. After allowing the cleavage reaction to proceed in cells, the conditioned media (secretome) and lysates were harvested and analysed by SDS-PAGE and immunoblotting (Fig. 2A). The cleavage of an RHBDL2 substrate, such as EGF, results in the accumulation of its proteolytic fragment in the culture medium, in some cases also accompanied by observable reduction in molecular weight of its transmembrane precursor in the cell lysate (Fig. 2A). Notably, we observed this pattern robustly for the candidate substrates BCAM, Spint-1, DDR1, CLCP1, Cadm1, and KIRREL, indicating they are genuine novel substrates of RHBDL2 (Fig. 2A). Some smearing of secreted fragments was observed in some cases, which was likely a result of extensive and/or variable glycosylation of the candidates’ extracellular domains, since it was alleviated or completely resolved by prior deglycosylation of the samples with PNGase F (asterisks). Interestingly, Fras1 appears to be processed by RHBDL2 in the cell lysate, but no corresponding proteolytic fragment can be found in the medium, even after PNGase F treatment (Fig. 2A). This may be the result of rapid internalization and/or degradation of the cleaved fragment, possibly relating to the fact that this is an extremely large protein and we had to use a truncated construct.

Importantly, we found a broad correlation between the H/L ratios found in the SILAC experiment and the intensity of cleavage seen in the immunoblotting experiments (Fig. 2A). For H/L ratios of ~0.72 and lower (as for IL6R), we verified that the majority of candidate proteins were indeed RHBDL2 substrates. For H/L ratios from 0.72 to 1.03, no cleavage was observed for the respective proteins. This was anticipated because an H/L ratio of 1 denotes that the secreted protein in question is at equivalent abundance in both SILAC samples independently of the presence of RHBDL2 activity. In summary, these experiments confirmed the accuracy and utility of SILAC proteomics approach for rhomboid substrate identification, and revealed several novel substrates of RHBDL2.

### RHBDL2 cleaves substrates at single sites after small amino acid residues within the N-terminal region of their TMD or in the immediate juxtamembrane region

To analyse the cleavage specificity of RHBDL2 and to generate uncleavable mutants as tools for further studies, we determined the sites of cleavage in the new substrates. We co-expressed the Strep-His tagged substrates with RHBDL2 in sufficient quantities to purify the N-terminal fragment from the media by sequential use of Ni-NTA and Streptactin resins. For MS analysis of the cleavage sites, the purified fragment was digested by trypsin or ArgC with the rationale that they should not interfere with the rhomboid cleavage sites because, based on the current knowledge^8, 17^, rhomboid proteases have different site specificity. Based on the identification of such most-C-terminal semitryptic or ‘semi-ArgC’ peptides in our novel RHBDL2 substrates, predicted to end in the P1 position of the RHBDL2-cleaved fragment, we mutated the candidate P1 residue(s) in the particular substrate to one that should prevent RHBDL2 mediated cleavage, such as phenylalanine, proline or threonine^8, 18^. Using this combined approach we identified cleavage sites by RHBDL2 in BCAM, Spint-1, CLCP1, KIRREL, DDR1 and IL6R (Fig. 2B), mutations of which blocked the RHBDL2-dependent shedding of these substrates (Fig. 2C). This means that each of these substrates was cleaved at a single site, located within or very close to the N-terminal (upper) portion of the transmembrane domain (TMD), and after a small amino acid (in the P1 position). The instances where a small amount of shedding of the P1 mutants could be observed are most likely due to high levels of substrate expression and residual cleavage of the mutant.

### The novel substrates are specific to RHBDL2

**Figure 3:**
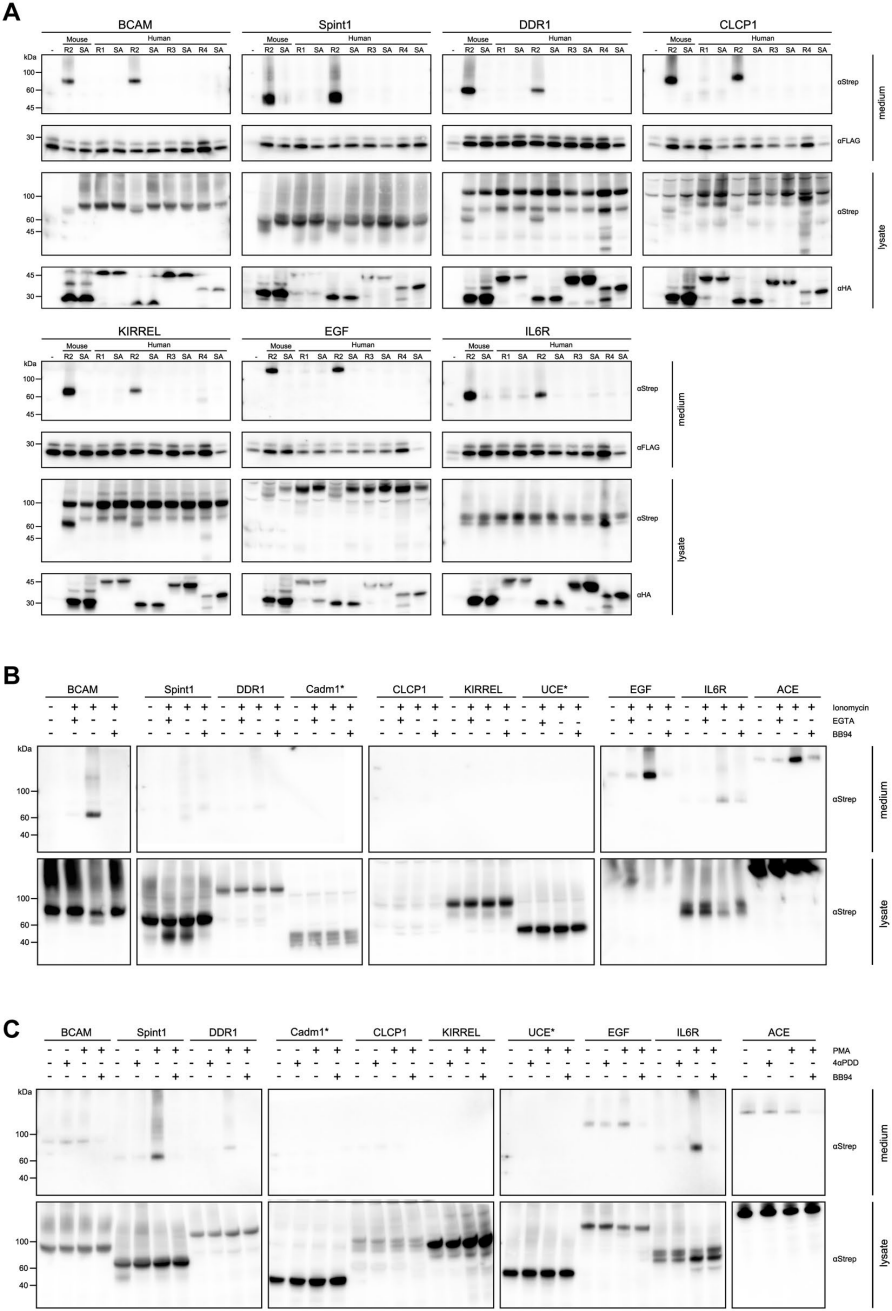
Identification of substrates specific for RHBDL2 (**A**). Strep tagged substrates were co-expressed with HA tagged forms of mouse RHBDL2, the four human rhomboids (R1/R2/R3/R4) and their corresponding inactivated forms where the catalytic serine residue was mutated to an alanine (SA). Twenty four hours after transfection, the medium was replaced by serum-free medium containing 10 µM broad spectrum matrix metalloprotease inhibitor BB94 to exclude shedding by these extracellular proteases. Forty eight hours after transfection, the media and cell lysates were harvested and analysed by immunoblotting. Cells were co-transfected with FLAG tagged prolactin as a secretion control and to confirm recovery of TCA precipitated proteins from the media. (**B**) Twenty four hours after transfection with plasmids encoding Strep tagged substrates, the medium was replaced with serum-free medium containing 1 µM ionomycin, 1 µM ionomycin and 2 mM EGTA, or 1 µM ionomycin and 10 µM BB94. One hour after media replacement the media (upper panels) and cell lysate (lower panels) were harvested as previously described and analysed by immunoblotting. (**C**) Twenty four hours after transfection with plasmids encoding Strep tagged substrates, the medium was replaced with serum-free medium containing 0.5 µM phorbol 12-myristate 13-acetate (PMA), the inactive analogue 4α-phorbol 12,13-didecanoate (4αPDD), or both 0.5 µM PMA and 10 µM BB94. One hour after media replacement the media and cell lysate were harvested as previously described and analysed by immunoblotting. Asterisks indicate samples which required treatment with PNGase F prior to SDS-PAGE to reduce smearing and improve resolution of bands on the gel.

We next focused on the most robust of the novel RHBDL2 substrates: BCAM, Spint-1, DDR1, CLCP1, KIRREL and IL6R, whose RHBDL2 cleavage sites we had been able to map. As mammalian genomes contain four secretase rhomboids, an obvious and important question was how selective these substrates are for RHBDL2, or whether there was any overlap in substrate repertoire with the other RHBDLs. We co-expressed the six most readily cleaved RHBDL2 substrates, plus EGF, with all four human secretase RHBDLs and assayed the effects on their processing and secretion. As shown in Fig. 3A, the N-terminal proteolytic fragments of all tested substrates can only be observed in the media upon co-expression with mouse or human RHBDL2, indicating that this is the sole RHBDL responsible for shedding of these proteins. As noted above, the secreted N-terminal fragments of the substrates can sometimes be also strongly observed in the cell lysate. This means that in some cases, cleavage happens not only at the plasma membrane, but also in intracellular compartments, presumably during trafficking of the enzyme and substrate to/from the cell surface. Interestingly, in the case of DDR1, KIRREL and CLCP1, we do observe some production of RHBDL4 dependent fragments in the cell lysate that are distinct from the pattern produced by RHBDL2 and which are not secreted. This is consistent with the proposed role of RHBDL4 in ER associated degradation^3^. Together, our data reveals that RHBDL2 exhibits a distinct specificity from other mammalian secretase rhomboids.

### Several RHBDL2 substrates are refractile to cleavage by ADAM10 or ADAM17

The functional relationship between intramembrane proteases and cell surface metalloproteases is an important consideration. For instance, the biology of gamma secretase is intrinsically linked to metalloproteases, because it only cleaves the stubs of cell surface proteins previously truncated by metalloproteases. By contrast, to date, evidence suggests that rhomboids do not require such prior truncation and appear to be capable of autonomous cleavage of their substrates. Nonetheless, several previous studies have noted the ability of metalloproteases to cleave rhomboid substrates, and metalloprotease inhibitors are often incorporated into rhomboid proteolysis experiments to prevent adventitious cleavage of rhomboid substrates by sheddases^8^. If a protein substrate is dependent on a particular protease rather than being promiscuously cleaved by several redundant enzymes, it is more likely that the dedicated protease will be relevant for the function of this protein. We therefore examined the relationship between RHBDL2 and metalloproteases for cleavage of the newly identified RHBDL2 substrates. As the previous experiments (Figs 1, 2 and 3A) were conducted in the presence of broad-spectrum metalloprotease inhibitor BB94, we next addressed whether any of our novel RHBDL2 substrates could also be cleaved by metalloproteases. In addition to comparing the shedding of RHBDL2 substrates with and without inhibiting metalloproteases, we went further and tested the impact of robust pharmacological stimulation of ADAM10 and ADAM17 on the shedding of RHBDL2 substrates.

The calcium ionophore ionomycin was used to elicit activation of ADAM10^19^. We expressed a range of our identified RHBDL2 substrates in HEK cells and harvested the media and cell lysates after treatment in the presence or absence of ionomycin (Fig. 3B). Epidermal growth factor (EGF) shedding has previously been shown to be largely dependent on ADAM10^12^ and we find that this protein is indeed shed upon ionomycin treatment (Fig. 3B, upper panels). Similarly, we observed that angiotensin converting enzyme (ACE) was constitutively shed by a BB94 sensitive metalloprotease activity (data not shown), and ACE shedding was strongly enhanced by ionomycin (Fig. 3B, upper panels) and blocked by EGTA and BB94. Of our identified substrates, only basal cell adhesion molecule (BCAM) and interleukin-6 receptor (IL6R) can be shed appreciably by activated ADAM10 (Fig. 3B, upper panels).

Activation of ADAM17 by phorbol-12-myristate-13-acetate (PMA)^19^ results in the shedding of a different subset of proteins from the cell surface (Fig. 3C, upper panels). In the presence of PMA, but not its inactive analogue, 4α-phorbol 12,13-didecanoate (4αPDD), only IL6R and Spint-1 (serine peptidase inhibitor, Kunitz type 1, also known as hepatocyte growth factor activator inhibitor 1, HAI-1) were appreciably shed, with some comparably minor shedding of DDR1 (discoidin domain receptor tyrosine kinase 1) visible (Fig. 3C, upper panels). This shedding was also sensitive to the broad spectrum metalloprotease inhibitor BB94. We conclude that the other novel RHBDL2 substrates tested (Cadm1, CLCP1, KIRREL and UCE) are completely resistant to the activity of both ADAM10 and ADAM17. Whilst this was not an exhaustive study of all known ADAMs, it does suggest that, as these proteins are not shed by the major metalloproteases at the cell surface, they may be entirely dependent on RHBDL2 for their shedding and biological signalling role. This emphasizes that, in contrast to gamma secretase, mammalian rhomboids can engage in signalling autonomously and that in cells expressing the two major ADAMs, several RHBDL2 substrates are spared by these sheddases.

### Endogenous RHBDL2 cleaves the newly identified substrates

**Figure 4:**
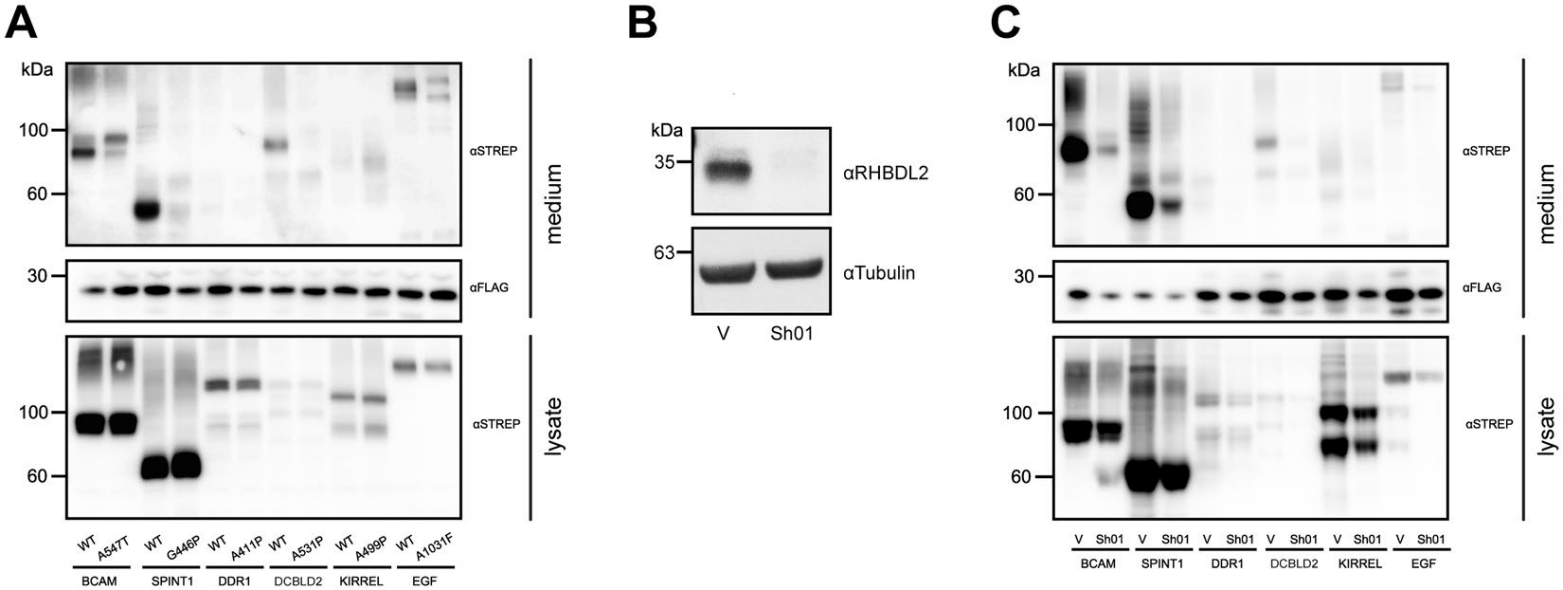
Endogenous RHBDL2 cleaves the newly identified substrates. (**A**) Strep tagged wild-type (WT) or P1-mutant substrates were expressed in HeLa cells, and shedding (in the presence of 10 µM BB94) was analysed as previously described. (**B**) RNA interference using stable expression of shRNA “01”^8^ specifically eliminates endogenous RHBDL2 protein in HeLa cells. Human RHBDL2 was visualised using a specific antibody (Protein Tech, 12467-1-AP at 1:500). V, pLKO.1 vector control; Sh01, specific shRNA targeted against the coding region of human RHBDL2^8^; (**C**) Strep tagged substrates were transfected into HeLa cells stably transduced with either empty vector pLKO.1 (V) or shRNA directed against RHBDL2 (Sh01)^8^. The cleavage assay was performed as previously described^8^.

To scrutinize the biological relevance of our novel substrates, we next investigated whether they are cleaved by endogenous RHBDL2 activity. To address this, we expressed the substrates in HeLa cells, which we previously showed to possess endogenous RHBDL2 activity^8^ (Fig. 4). In this cell line, the ectodomains of BCAM, Spint-1, and CLCP1 were visibly shed into the media and this activity was abolished by mutation of the P1 residue at the RHBDL2 cleavage site, emphasizing the dependency on RHBDL2 (Figs 2B and 4A). In addition, stable suppression of RHBDL2 by RNA interference using a validated specific shRNA^8^ (Fig. 4B) also abolished the secretion of BCAM, Spint-1, and CLCP1 ectodomains, while transducing HeLa cells stably only with the vector control had no effect (Fig. 4C). In both contexts, we were unable to detect proteolytic release of DDR1 and KIRREL despite them being strongly cleaved by RHBDL2 in the overexpression assay. This may be simply a result of reduced endogenous RHBDL2 activity in HeLa cells, or, alternatively, it might indicate that a stimulus may be required for the RHBDL2-catalyzed shedding of these substrates in an endogenous setting. Overall, the fact that overexpressed RHBDL2 can cleave endogenous substrates (our original SILAC experiment) and that, in an independent context, endogenous RHBDL2 cleaves several overexpressed substrates, collectively indicates that RHBDL2-mediated cleavage of these proteins at the cell surface is a physiologically relevant phenomenon.

### Expression profile and identity of RHBDL2 substrate repertoire suggest functions in epithelial homeostasis

**Figure 5:**
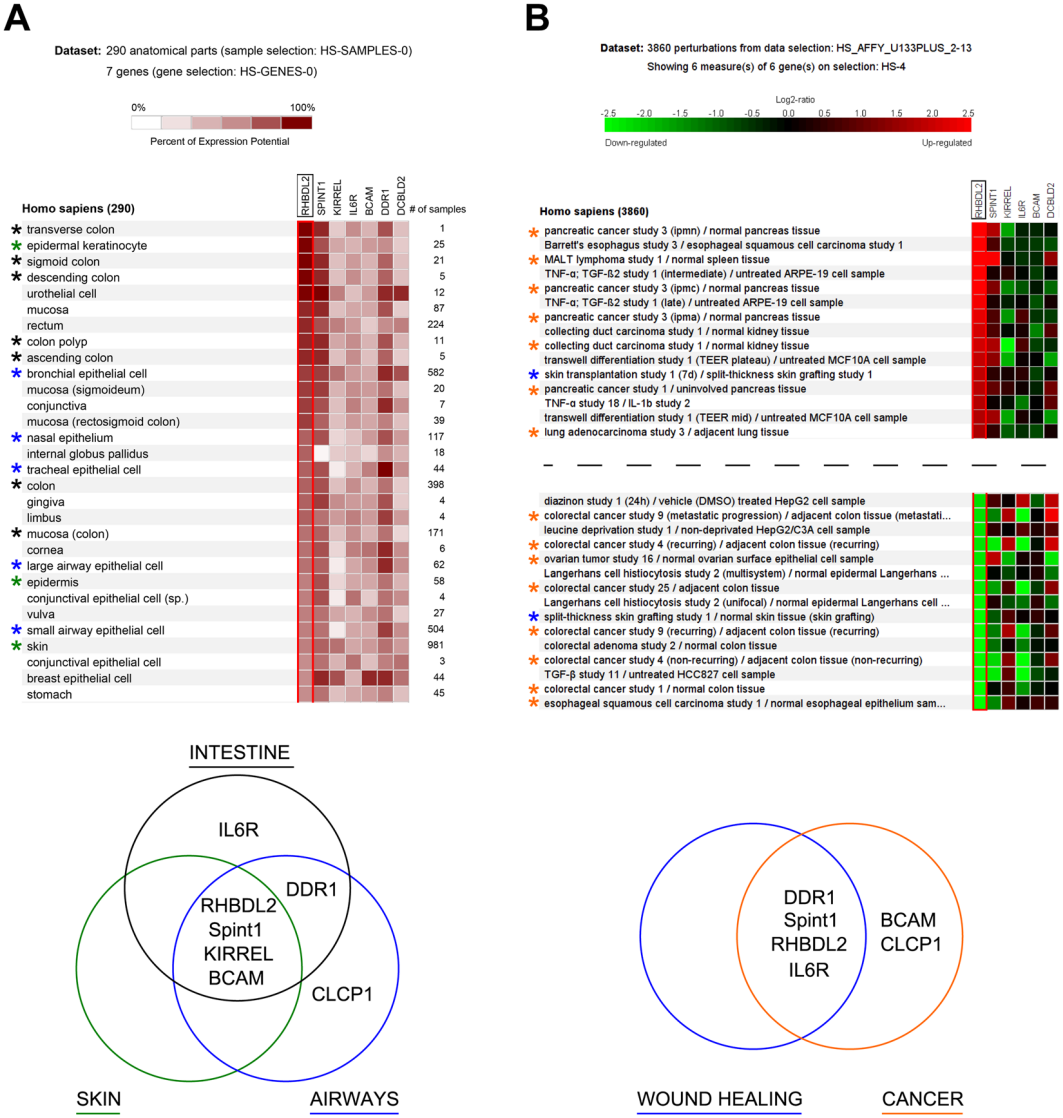
Overlapping functions and expression of novel RHBDL2 substrates. Gene expression was analysed using the Genevestigator environment^20^. See main text for further details. (**A**) Co-expression of RHBDL2 and its substrates. Tissues were ranked from high to low according to the relative expression level of RHBDL2 and the top 30 results are displayed. The relative expression level of novel substrates was also analysed in these tissues and functionally linked tissues are highlighted with colour-coded asterisks and summarised in the Venn diagram which shows particularly high levels of expression of RHBDL2 and its substrates occurs in distinct tissues. (**B**) Potential biological roles for RHBDL2 and its substrates. Studies were ranked according to conditions in which RHBDL2 is both most upregulated and downregulated and the top 15 results in each case are displayed. Regulation of novel RHBDL2 substrates is presented alongside. Recurring conditions are highlighted with colour-coded asterisks and summarised in the Venn diagram which shows changes in gene expression of RHBDL2 and its substrates occurs in distinct biological processes.

To obtain further insight into the physiological relevance of the RHBDL2 substrates, we investigated the expression of their mRNAs and their potential co-expression with RHBDL2 in human tissues using the Genevestigator platform of manually curated mRNA expression data^20^. Strikingly, we found that RHBDL2 is strongly expressed in epithelial tissues, particularly those of the digestive tract, skin and airways (Fig. 5A), and the expression of Spint-1 matches this pattern extremely well. Moreover, other substrates are co-expressed with RHBDL2 highly in specific tissues, such as DDR1 in the colon, urothelial cells and bronchial epithelial cells, and CLCP1/DCBLD2 in the urothelial cells and bronchial epithelial cells. These tissues are all exposed to the external environment and are therefore most likely to encounter physical injury.

Interestingly, RHBDL2 and several of its newly identified substrates have been previously implicated in wound healing^21–24^, and using the same expression dataset we identify strong modulation of RHBDL2 gene expression in skin grafting and transplantation studies where epithelial wound healing must be regulated (Fig. 5B). The wound healing process also requires a strict regulation of cell migration to seal wound margins, the dysregulation of which is a hallmark of cancer. We observe substantial variation in the gene expression of RHBDL2 and the substrates we identify here in several cancer studies, particularly in RHBDL2-rich tissues such as the skin, airways and digestive tract (Fig. 5B). Taken together, these data indicate that RHBDL2 catalyzed cleavage of Spint-1, DDR1 and CLCP1/DCBLD2 might play (patho)physiological roles in the skin, airways and digestive tract. Considering also the described functions of the identified substrates BCAM, Spint-1, DDR1, Cadm1, KIRREL, CLCP1 and IL6R, which involve mainly cell adhesion and migration (Fig. 5B), we propose that RHBDL2 functions in epithelial homeostasis.

## Discussion

In this study we developed and applied a quantitative proteomics technique to identify the substrate repertoire of the mammalian rhomboid protease RHBDL2. The proteomics screen was limited to a single biological replicate, but we extensively verified that the majority of the candidate hits identified by the screen are bona fide RHBDL2 substrates, validating the approach. Several novel and potentially generalizable premises emerge from this objective screen for substrates of a rhomboid protease. First, the majority of the novel RHBDL2 substrates identified are type I membrane proteins, indicating that RHBDL2 selectively sheds this topology of membrane proteins from the plasma membrane. Several previous studies have come to the same conclusion^2, 7, 9^, albeit focusing on a much smaller number of specific candidate substrates. A second conclusion is that RHBDL2 is a relatively prolific protease. If we restrict our calculations to type I membrane proteins, representing 20% of the protein cohort detected in our study (Fig. 1B), this indicates that RHBDL2 cleaves around 15% (13 out of 91) of all type I membrane proteins that our study could detect. This observation must be tempered by factoring in the biological relevance and relative importance of RHBDL2 cleavage of all of these novel substrates, which can only be addressed by subsequent studies.

Another important principle revealed by our work is the finding that the substrates are specific to RHBDL2 and other mammalian RHBDLs cannot shed them. This is objective evidence that mammalian rhomboids exhibit distinct substrate specificities. As mammalian rhomboids localize to a range of vesicular compartments, further studies will be required to reconcile whether their substrate preferences are governed by proteolytic specificity, and/or compartmentalization. It is also notable that several of our substrates are spared by the major metalloprotease sheddases, ADAMs 10 and 17. Our data highlight that a subset of proteins is entirely dependent upon RHBDL2 for their proteolytic release from the membrane. This finding helps to reconcile the relationship between rhomboids and metalloproteases, illustrating, in contrast to previous studies^2, 7, 8^ that RHBDL2 does not simply serve a redundant function in shedding substrates that are shared with other cell surface proteases. It further suggests that intramembrane cleavage of these substrates by RHBDL2 may be integral to their biological functions.

Previous studies on RHBDL2 have proposed several potential biological functions for it. RHBDL2 mediated cleavage of thrombomodulin has previously been shown to facilitate wound healing by promoting cell migration of epithelial keratinocytes to seal the wound margin^21^. Cleavage of another substrate, CLEC14A, modulates sprouting angiogenesis in endothelial cells^9^. In addition, overexpression of RHBDL2 in epithelial cells has been suggested to promote resistance to anoikis, a process which drives apoptosis in detached cells and is important for preventing metastasis in cancer^25^. All these processes require careful regulation of cell adhesion, migration and sensing of the extracellular environment^26–28^. These features are relevant to a number of diseases connected with impaired epithelial homeostasis, such as chronic epithelial injury, cancer, inflammation and fibrosis. Our study supports these central themes on the basis of an objectively determined and substantially expanded substrate repertoire. Analysis of the gene expression of RHBDL2 and our most rigorously characterised substrates highlights that they are highly expressed in epithelia of the skin, airways and digestive tract and exhibit changes in their expression under similar pathological conditions. Some of the substrates we identify can also be linked to the maintenance of epithelial homeostasis based on current literature. For example, Spint-1 is an inhibitor of several cell surface proteases involved in growth factor activation^29^ and its expression is elevated in conditions of chronic wounding^22^, liver fibrosis^30^ and lung fibrosis^31, 32^. Discoidin domain receptor 1 (DDR1) is a receptor tyrosine kinase that is activated by collagen, resulting in phosphorylation of the intracellular domain and signalling cascades that regulate cell migration, proliferation and extracellular matrix remodelling through matrix metalloproteases^33, 34^. Determining the consequences of RHBDL2 mediated cleavage of such substrates will be an important challenge for the future, considering that ectodomain shedding of cell surface proteins by ADAMs and RHBDL2 has been demonstrated to be either activating or inhibitory for certain processes depending on the identity of the substrate^9, 21, 35^.

In conclusion, our study demonstrates several important advances. We establish a methodology to identify objectively the repertoire of substrates of a mammalian rhomboid protease. Our approach reveals that mammalian rhomboids target, in a non-redundant way, a range of cellular substrates that are thematically related and may be implicated in fundamental biological and disease-related processes, and that some of these substrates are refractile to cleavage by ADAM proteases. Such information can be used to build a hypothesis concerning the biological function of rhomboid proteases that can subsequently be investigated in mutant mouse models. Our approach can now be applied where it is acutely needed, in particular to the orphan mammalian rhomboid proteases RHBDL1 and 3. More broadly, our work emphasizes the importance of studying, in more detail, the cellular and organismal roles of mammalian rhomboid proteases and their substrates, by objective approaches.

## Methods

### Antibodies and reagents

Anti-Strep antibody was obtained from Qiagen, anti-HA-HRP and anti-FLAG-HRP were supplied by Roche and Sigma-Aldrich, respectively. Endogenous human RHBDL2 was visualised by the 12467-1-AP antibody (ProteinTech) at 1:500, and tubulin by the sc-8035 antibody (Santa Cruz) at 1:2000.

HRP-conjugated secondary antibodies were from Santa Cruz biotech. Phorbol-12-myristate-13-acetate (PMA), 4α-phorbol 12,13-didecanoate (4αPDD) and ionomycin were purchased from Merck Millipore.

### DNA cloning and constructs

The lentiviral expression construct encoding untagged mouse EGF, shRNAs specific to human RHBDL2, and the expression construct for mouse RHBDL2, tagged with a triple N-terminal HA tag in pcDNA3.1, were all described previously^8^. Triple N-terminally HA-tagged human RHBDL2 has been described^2^ and RHBDL 1, 3 & 4 (IMAGE 40081042, 40005244 and 40023929 respectively) were cloned into the same vector in-frame with a triple N-terminal HA-tag. The catalytic serine in each RHBDL construct (S312, S187, S278 and S247 respectively) was mutated to alanine by site-directed mutagenesis. For the production of lentiviruses expressing WT RHBDL2 or its catalytically inactive S186A derivative, the coding sequences of mouse RHBDL2 WT or SA mutant, containing an N-terminal triple HA tag, were inserted into the EcoRI site of the lentiviral expression vector pTetO-FUW-Klf-4 (Addgene plasmid 20322)^36^. The resultant plasmids were used, in the absence of expression of the Tet repressor, to achieve stable expression of RHBDL2 or its mutant. The cDNAs encoding candidate substrates have been obtained from various sources, as IMAGE clones or from academic labs based on published constructs. The substrate ORFs or their fragments (as specified in Table 1) were cloned into a pcDNA3.1 derived vector encoding a Drosophila Spitz signal peptide followed by Twin-Strep and His tags and fully sequenced.

### Cell line construction and SILAC labelling

HEK293ET cells were stably transduced with lentiviruses expressing mouse EGF^8^, then split in two subcultures that were stably transduced with lentivirus encoding either wild type RHBDL2 or its catalytically dead mutant (RHBDL2.S186A). At this point, cells were cultured in DMEM medium (Invitrogen) supplemented with 10% foetal calf serum (Thermo) at 37 °C and 10% CO_2_. The media for the SILAC labelling were composed of DMEM medium -Arg -Lys (Invitrogen, #88420), dialysed foetal calf serum (Thermo Scientific, #26400-044), supplemented with 0.798 mM L-Lys (Sigma L9037) and 0.398 mM L-Arg (Sigma A6969) in the light condition, or 0.798 mM ^13^C_6_-^15^N_2_-L-Lys (Sigma-Isotec 608041) and 0.398 mM ^13^C_6_-^15^N_4_-L-Arg (Sigma-Isotec 608033) in the heavy condition. For SILAC labelling, cells expressing wild type RHBDL2 were grown in the presence of light L-Arg and L-Lys while cells expressing RHBDL2.S186A were grown in the presence of heavy L-Arg and L-Lys. Cells were allowed to grow in the SILAC media for approximately 8-10 doublings to a scale of 10 × 10 cm dish per condition, at which point they were washed in serum-free SILAC medium and then incubated in serum-free light or heavy SILAC DMEM (6 ml per dish) plus 10 µM metalloprotease inhibitor BB-94, and cultured for further 26 hrs. After this time, culture supernatants were harvested and mixed 1:1 by volume, centrifuged to remove any remaining cells and debris, filtered through a 0.22 µm polyethersulfone (PES) syringe-filter, and concentrated using Vivaspin 15R (2 kDa MWCO, Sartorius). Glycoproteins were enriched by wheat-germ agglutinin (WGA) and concanavalin A (ConA) Glycoprotein Isolation Kits (Pierce).

### Mass spectrometry and data analysis

The glycoprotein-enriched mixed light and heavy media were precipitated using 15%(w/v) trichloroacetic acid (TCA), collected by centrifugation, washed with acetone, and the pellet was stored at −70 °C. Protein samples for mass spectrometry were prepared for analysis using a filter-aided sample preparation method, essentially as described previously^37^. Briefly, the protein sample was reduced in a buffer containing 8 M urea, 0.1 M Tris-HCl, pH 8.5, 10 mM DTT, and 0.05%(w/v) Rapigest (Waters). The protein mixture was divided equally between four Amicon Ultra 0.5 ml centrifugal filter units (EMD Millipore) and then alkylated with 55 mM iodoacetamide. After alkylation the proteins were buffer exchanged into 8 M urea, 0.1 M Tris-HCl, pH 8. Proteins were digested on the membrane for 18 h at 37 °C with 800 ng endoproteinase Lys C (Roche). The sample was subsequently digested with 800 ng trypsin (Promega) for 4 h at RT. The filter units were then centrifuged at 14,000× *g* for 20 min and the peptide filtrates pooled, de-salted using a Waters Sep-Pak Light C_18_ cartridge and lyophilized overnight.

Peptides were then reconstituted in a buffer containing pH 3–10 ampholytes and resolved into 12 fractions using an Agilent 3100 Off-Gel fractionator on 13 cm, pH 3–10 Immobiline gel strip (GE Healthcare). The fractions generated were desalted with 3 M C_18_ in house stage tips and lyophilized to dryness.

The peptides were reconstituted in 200 µl of 3%(v/v) acetonitrile/0.1%(v/v) formic acid and 10 µl of this solution was analyzed by nano-scale capillary LC-MS/MS using an UltiMate U3000 HPLC (Thermo Fisher Scientific) to deliver a flow of 200 nl/min. A µ-precolumn cartridge, C_18_ Acclaim PepMap 100 (5 µm, 300 µm × 5 mm; Thermo Fisher Scientific), trapped the peptides before separation on a C_18_ Acclaim PepMap100 (3 µm, 75 µm × 250 mm; Thermo Fisher Scientific). Peptides were eluted with a 110 minute gradient of acetonitrile (5–40% (v/v)). The analytical column outlet was directly interfaced via a modified nano-flow electrospray ionization source, with a hybrid linear quadrupole ion trap mass spectrometer (Orbitrap LTQ XL; Thermo Fisher Scientific). Data-dependent analysis was performed using a resolution of 60,000 for the full MS spectrum, followed by 10 MS/MS spectra in the linear ion trap. MS spectra were collected over an m/z range of 350 to 2,000. MS/MS scans were collected using threshold energy of 35 for collision-induced dissociation.

The raw mass spectrometric data files for each fraction were processed and analysed using MaxQuant (v1.5.2.8). The search was performed using a Uniprot/Swissprot human database containing protein isoforms (downloaded 17/03/03) with common contaminants included. Enzyme specificity was set to trypsin, with variable modifications, methionine oxidation and protein *N*-acetylation. Cysteine carbamidomethylation was considered a fixed modification. Heavy labels were set to R10K8. An MS/MS tolerance of 0.5 Da, a minimal peptide length 6, and 2 missed cleavages were allowed. Proteins were considered identified if they had at least one unique peptide, and quantified if they had at least one quantifiable SILAC pair. The resulting MaxQuant^27^ protein table was processed using Perseus 1.5.6.0^28^ implementing Phobius^15^ topology predictions (see Table S1). The quantified peptides were graphically mapped to the corresponding protein sequences using the Proteator tool (http://proteator.appspot.com/)^38^ with I/L nondifferentiation turned on; the html overview of the results can be found in the Supplementary Information. The mass spectrometry proteomics data have been deposited to the ProteomeXchange Consortium (http://proteomecentral.proteomexchange.org) via the PRIDE partner repository^39^ with dataset identifier PXD006716.

### RHBDL2 activity assay in cell culture

All cells were cultured in Dulbecco’s Modified Eagle’s Medium (DMEM) supplemented with 10% foetal calf serum. The day after plating in either 6 or 24 well plates, cells were transfected with RHBDL2 substrates, RHBDL2, FLAG tagged prolactin (as a secretion control) and GFP plasmid DNA, to a total of 1 µg, using ExtremeGene 9 transfection reagent (Roche) according to manufacturer’s instructions. As the substrates expressed with varying efficiencies, we adjusted the amount of transfected DNA so that the expression levels of all substrates were normalized. Approximately 24 hr post-transfection, cells were washed with PBS and incubated for 24 hours in serum free DMEM containing 50 U/ml penicillin, 50 µg/ml streptomycin, 10 µM BB94 and any treatments as stated. Media was harvested and centrifuged at 20000× *g* to remove cell debris prior to precipitation with 15% (w/v) TCA. Precipitated protein was pelleted by centrifugation and washed with acetone to remove TCA before solubilisation in 30 µl (6 well plate) or 15 µl (24 well plate) SDS-PAGE sample buffer. Cell monolayers were harvested directly in 150 µl (6 well plate) or 30 µl (24 well plate) SDS-PAGE sample buffer supplemented with 20 mM MgCl_2_ and 50 U/ml benzonase (Novagen) to reduce sample viscosity. To improve resolution by SDS-PAGE, some samples were subsequently treated with PNGase F (NEB) for 1 hr at 37 °C according to manufacturer’s instructions.

### Identification of RHBDL2 cleavage sites

Rhomboid cleavage assays were scaled up to a T75 flask format and 70 ml media from 5 flasks expressing RHBDL2 and the substrate of interest was pooled, filtered through a 0.22 µm syringe-filter to remove cell debris and supplemented with a protease inhibitor cocktail tablet (Roche). The media was concentrated using Vivaspin centrifugal concentrators (PES membrane, MWCO 10 kDa) to a final volume of 5 ml. The media was supplemented by 0.5 ml NiNTA buffer base (0.5 M NaH_2_PO_4_, 1.5 M NaCl, 100 mM imidazole, pH 8.0) and incubated with 0.5 ml NiNTA agarose pre-equilibrated with NiNTA loading buffer (500 mM NaH_2_PO_4_, 300 mM NaCl, 10 mM imidazole, pH 8.0) for one hour at 4 °C with agitation. The mixture was loaded into a gravity flow column, washed four times with NiNTA loading buffer and eluted with two 1 ml volumes of NiNTA elution buffer (500 mM NaH_2_PO_4_, 300 mM NaCl, 250 mM imidazole, pH 8.0). Eluates were pooled and incubated with 50 µl StrepTactin agarose (IBA) pre-equilibrated with 50 mM Tris-HCl, pH 8.0 for one hour at 4 °C with agitation. The mixture was then transferred to a microspin column (Pierce) and washed three times with 50 mM Tris-HCl, pH 8.0. The protein was eluted in three 50 µl volumes of 2.5 mM desthiobiotin, 50 mM Tris-HCl, pH 8.0. The eluates were pooled and incubated with two units PNGase F at 37 °C for 3 hr to remove glycans. The resulting sample was analysed by mass spectrometry to identify the most C-terminal semitryptic or semi-ArgC peptide(s).

### Pharmacological stimulation of ADAM protease shedding activity

The assay to monitor shedding of substrates by pharmacologically activated ADAM proteases (Fig. 3B and C) was set up in a similar manner to the standard RHBDL2 cleavage assay with the following modifications: 24 hr after transfection, the cells were washed with PBS and incubated for 1 hr in serum free DMEM containing 50 U/ml penicillin, 50 µg/ml streptomycin and any stated treatments. Cells were treated with ionomycin or PMA, as indicated in the figure legends, to stimulate the sheddase activity of ADAM 10 or 17, respectively. Media and cell lysates were harvested and immunoblotted as described above.

### Lentiviral transduction of RHBDL2 shRNAs

The human RHBDL2-specific shRNAs were described previously^8^. To generate the lentivirus, 2.6 µg of pLKO.1 plasmids encoding an shRNA targeted against human RHBDL2 (sh01) or empty vector were co-transfected alongside 1.8 µg pCMVΔ8.91 and 0.78 µg pMD-VSVG into HEK cells using ExtremeGene 9 transfection reagent (Roche). The viral particles were allowed to accumulate in the media for approximately 48 hr and then filtered through a 0.45 µm PES membrane, diluted fourfold with DMEM and supplemented with 10 µg/ml Polybrene. Target HeLa cells were incubated with this viral solution for 24 hr at 37 °C and then selected by exchanging the medium for DMEM supplemented with 2.5 µg/ml puromycin.

### Western Blotting

Ten µl of each sample were loaded onto 7.5%, 12% Tris-glycine SDS-PAGE gels or 4–20% gradient gels (Biorad) depending on the molecular weight of the proteins of interest. Proteins were transferred to PVDF membranes and subsequently blocked with casein blocker (Thermo Scientific) or non-fat milk (Cell Signalling Technologies) for 1 hr at room temperature. Membranes were probed with primary antibody overnight at 4 °C and following washing steps were incubated with the appropriate HRP-conjugated secondary antibody for 2 hr at room temperature. The signal was detected using a CCD camera and Luminata Crescendo (Merck Millipore) or West Femto (Pierce) chemiluminescent HRP substrate depending on the intensity of the signal.

### Data Availability

The proteomics dataset presented in this article is available free of charge at the ProteomeXchange repository under the identifier PXD006716 (www.proteomexchange.org).

## Electronic supplementary material

Supplementary Information
