# Supplementary material for: Quantitative proteomics screen identifies a substrate repertoire of rhomboid protease RHBDL2 in human cells and implicates it in epithelial homeostasis
Source: Sci Rep. 2017 Aug 4;7:7283. doi: 10.1038/s41598-017-07556-3 (PMC5544772; doi:10.1038/s41598-017-07556-3)
Supplement: Supplementary file 1 — Supplementary Information [file 41598_2017_7556_MOESM1_ESM.pdf]

Supplementary Information

Quantitative proteomics screen identifies a substrate repertoire of rhomboid protease RHBDL2 in human cells and implicates it in epithelial homeostasis.

[1]Nicholas Johnson, [1,2]Jana Březinová, [3]Elaine Stephens, [5]Emma Burbridge, [3,4]Matthew Freeman, [5,]Colin Adrain* and [1,]Kvido Strisovsky*

[1]Institute of Organic Chemistry and Biochemistry, Czech Academy of Science, Flemingovo n. 2, Prague, 166 10, Czech Republic

[2]Department of Biochemistry, Faculty of Science, Charles University, Prague, Czech Republic

[3]MRC Laboratory of Molecular Biology, Cambridge, CB2 2QH, United Kingdom

[4]Sir William Dunn School of Pathology, Oxford, OX1 3RE, United Kingdom

[5]Instituto Gulbenkian de Ciência, Lisbon, Portugal

*Authors for correspondence (email: kvido.strisovsky@uochb.cas.cz, cadrain@igc.gulbenkian.pt)

Table S1: The full dataset of proteins identified in the SILAC experiment.

The table summarizes all proteins with at least one quantifiable SILAC pair identified by MaxQuant[15] with corresponding topology predictions by Phobius[17].

Table S1 rev1.xlsx

Please open attachment from the sidebar.

Dataset S1: Results of proteomics experiment to identify RHBDL2 substrates (Proteator format).

Proteator results Johnson et al.html

Please open attachment from the sidebar.
